# Robust Human Activity Recognition by Integrating Image and Accelerometer Sensor Data Using Deep Fusion Network

**DOI:** 10.3390/s22010174

**Published:** 2021-12-28

**Authors:** Junhyuk Kang, Jieun Shin, Jaewon Shin, Daeho Lee, Ahyoung Choi

**Affiliations:** 1 Department of Software, Gachon University, Seongnam 13120, Korea; wnstm0130@gmail.com (J.K.); xeaquz@gmail.com (J.S.); pq1031@gmail.com (J.S.); 2 Department of Mechanical Engineering, Gachon University, Seongnam 13120, Korea; dhl@gachon.ac.kr

**Keywords:** human activity recognition, deep learning, fusion network, accelerometer sensors, skeleton detection

## Abstract

Studies on deep-learning-based behavioral pattern recognition have recently received considerable attention. However, if there are insufficient data and the activity to be identified is changed, a robust deep learning model cannot be created. This work contributes a generalized deep learning model that is robust to noise not dependent on input signals by extracting features through a deep learning model for each heterogeneous input signal that can maintain performance while minimizing preprocessing of the input signal. We propose a hybrid deep learning model that takes heterogeneous sensor data, an acceleration sensor, and an image as inputs. For accelerometer data, we use a convolutional neural network (CNN) and convolutional block attention module models (CBAM), and apply bidirectional long short-term memory and a residual neural network. The overall accuracy was 94.8% with a skeleton image and accelerometer data, and 93.1% with a skeleton image, coordinates, and accelerometer data after evaluating nine behaviors using the Berkeley Multimodal Human Action Database (MHAD). Furthermore, the accuracy of the investigation was revealed to be 93.4% with inverted images and 93.2% with white noise added to the accelerometer data. Testing with data that included inversion and noise data indicated that the suggested model was robust, with a performance deterioration of approximately 1%.

## 1. Introduction

Behavior recognition technologies are increasingly being employed in wearable-based fitness trackers as user interest in health grows. Because most wearable devices have an acceleration and gyro sensor, sensor signals can be used to recognize behavior. Simple actions such as walking, running, and running can be analyzed while wearing a watch. In line with this trend, various studies are being actively conducted to analyze and recognize behavior patterns using smart devices, such as mobile phones and wearable devices [1,2,3,4]. Some researchers have investigated the accuracy of behavior recognition based on the position of a wearable sensor. The most optimal sensor placement for actual behavior identification is the waist, ankle, or hip, despite most wearable devices being in the form of a watch worn on the wrist [3,4]. Stewart et al. used a logistic regression model to segment a 10 s windowed acceleration signal from the hip and wrist and found that the hip sensor achieved an accuracy of 91%, whereas the wrist sensor had an accuracy of 88.4% [4].

The acceleration signal was used as an input signal to extract features and then used as an input for the deep learning model. Methods for extracting the characteristics of the accelerometer signal include applying the mean, median, zero-crossing, correlation, and signal vector magnitude. The extracted features are used as inputs for machine learning and deep learning models to recognize behavior patterns. In a study conducted by Anahita et al., the accelerometer and gyroscope data of 25 children were measured using a smart watch [5]. Children wore smartwatches and conducted six different activities. Their running, walking, standing, sitting, lying, and stair climbing were measured at 10 Hz at 10 min intervals. The measured data were subjected to a preprocessing process suitable for each feature type, and features such as the mean, median, FFT-entropy, and signal vector magnitude were extracted and used as inputs for the deep learning model. Six activities were classified using two deep learning techniques: DNN and RNN. The RNN model showed an average F1 score of 80%.

Ahmadi et al. used machine learning techniques to recognize the behavioral patterns of adolescents and children with cerebral palsy into four classes: sedentary (SED), standing utilitarian movements (SUM), comfortable walking (CW), and brisk walking (BW) [6]. They used the signal vector magnitude feature extracted from 10 s non-overlapping segmented signals, which were measured from 22 children and adolescents. Using the extracted features, four classes were classified using three machine learning techniques: random forest (RF), support vector machine (SVM), and binary decision tree (BDT). The results showed that the SVM and RF performed better than the BDT, with an average of 82.0% to 89% for the SVM, 82.6% to 88.8% for the RF, and 76.1% to 86.2% for the BDT. By class, SED was 94.1% to 97.9%, SUM was 74.0% to 96.6%, CW was 47.6% to 70.4%, and BW was 71.5% to 86.0%, which showed a good performance in the SED and SUM classifications, but a poor performance in the CW and BW classifications. Ignatov recognized the acceleration signal based on a user-independent CNN model and analyzed it using the UCI database, in which six types of behavioral data, including jogging, walking, and climbing stairs, were collected [7]. Wang et al. recognized 19 behaviors using an auto-encoder composed of a deep belief network in unsupervised learning based on signals collected through wearable devices and showed a performance of 99.3% [8].

Unlike in previous studies, there is a trend of applying deep learning models based on raw data without preprocessing [9,10]. It has been reported that a deep neural network model performs better by finding more information than a shallow neural model [11]. Existing studies have used only accelerometer data or extracting features from the sensor data; however, the model can find more usable features than humans are unable to find. However, if a preprocessing is not applied, the model can be trained with better features because it learns by extracting features directly from the raw data [12]. River et al. proposed an RNN-based human activity recognition model to classify six hand activities [13]. They used inertial sensor data directly in the proposed model without preprocessing. Zhao et al. proposed a customized long short-term memory (LSTM) model by varying the window size in the data segmentation step [14]. They used an accelerometer, a gyroscope, and magnetometer sensors as inputs without preprocessing. They found the optimized window size to obtain improved results; their final recognition accuracy was 93.6% using the UCI HAR database. Hassan et al. presented an end-to-end deep neural network (DNN) model for recognizing human actions from temporally sparse data signals generated by passive wearable sensors [15]. Wan et al. proposed an HAR architecture based on a smartphone inertial accelerometer [16]. The smartphone gathered the sensory data sequence while the participants went about their everyday activities and extracted the high-efficiency features from the original data through numerous three-axis accelerometers. To extract the relevant feature vectors, the data were preprocessed through denoising, normalization, and segmentation. They applied CNN, LSTM, BLSTM, MLP, and SVM models using the UCI and Pamap2 datasets and observed 93.21% accuracy with the CNN model using the UCI dataset.

In addition to the accelerometer signal, research on recognizing user behavior based on image signals is being actively conducted. In the case of images, studies are actively being applied to extract image features using a scale-invariant feature transform (SIFT) or speeded-up robust features, and to predict poses by recognizing silhouettes, depth information, and skeletons. Kale et al. proposed video-based human activity recognition for a smart surveillance system [17]. The system extracted the features based on SIFT and applied a K-nearest neighbor (KNN) and an SVM to recognize four to nine activities including falling, fighting, walking, running, and sitting, among other general actions. The results show that an SVM achieves a 92.91% accuracy rate, whereas a KNN has an accuracy rate of 90.83%. Kim et al. proposed the activity recognition of elderly people using skeleton joint features from a depth video [18]. They applied a hidden Markov model to distinguish between diverse human behaviors. The results of the experiments demonstrate that the elderly achieve a higher recognition rate, with a mean recognition rate of 84.33% for nine daily regular activities.

In recent years, the accuracy of behavior recognition has been significantly improved by applying deep learning and machine learning technologies. Khaire et al. applied a 5-CNN model using various vision cues, such as RGB images, depth images, and skeletal data as inputs. The performance of the 5-CNN model was 95% to 96% for classifying 27 activities, including bowling, boxing, tennis, and swinging [19]. In addition, in self-supervised learning, a small number of data is augmented, and a study based on rotation data is being conducted. However, there is a problem in that it is difficult to recognize whether the image has been rotated, and thus whether lying or standing can be recognized as different poses. Amir et al. classified 60 classes using NTU RGB-D data as the input of the proposed 2-layer Part-Aware LSTM model [20]. The class contains 40 daily actions (e.g., drinking, eating, and reading), nine health-related actions (e.g., sneezing, staggering, and falling), and 11 mutual actions (e.g., punching, kicking, and hugging). It was confirmed that the proposed model showed a cross-subject accuracy of 62.93% and a cross-view accuracy of 70.27%. Because of the complexity of human activity sequences, Shahroudy et al. suggested a multimodal multipart learning method that supports the sparse combination of multimodal part-based characteristics using depth and skeleton data [21].

Research on integrating multiple heterogeneous sensory information is being conducted. Some existing studies were conducted to recognize behavior by extracting and integrating various feature values, such as silhouette and depth information, from the video signal. Khaire et al. proposed a method integrating vision data such as RGB, silhouettes, and skeletons [19]. Amir et al. proposed RGB and depth data to recognize human activities [20]. In addition, research integrating various sensor data such as accelerometers, gyroscopes, and magnetic field signals to recognize behavior have been conducted [22]. Wei et al. proposed a CNN-based deep learning model to integrate the video and inertial-sensing signal in order to detect human activities [23]. In this research, continuous motion was expressed using a three-dimensional video volume and an input translated from a one-dimensional acceleration signal into a two-dimensional image form using a spectrogram. CNN was employed in the behavior recognition model, as well as two types of fusion models. Fusion was performed at the decision level in the first model following classification for each input, while fusion was performed at the feature extraction level in the second model. The fusion at the feature level was 94.1% accurate, and the fusion at the decision level was 95.6% accurate.

Recently, much research has been undertaken to examine performance according to the enhanced method of distinguishing the backdrop from the person in the image, data segmentation, feature extraction, and feature selection, in order to increase the accuracy of behavior identification. Kiran et al. proposed a deep learning model with five multi-layers based on CNN that optimizes computation time [24]. Each deep learning model was used for database normalization, transfer-learning-based optimal feature extraction, fusion and classification, and the Shannon entropy theory, a statistical feature, was applied to feature selection. In this study, by applying various databases such as UCF sport, KTH, and UT interaction, it was verified through experiments whether there was an improvement in processing speed while maintaining accuracy. Khan et al. proposed a cascaded framework for action recognition from video sequences [25]. They used frame enhancement by contrast stretching, luminance channel selection, and so on, to clearly distinguish between the background and the person. After classifying it using the removed background and saliency map, a morphological operation to classify the human form was derived. Then, various types of features such as HOG and SFTA were extracted from the image, fused, and then classified by applying a neural network. The proposed method showed 97.2% to 99.9% performance in various open databases, such as KTH, UIUC, Muhavi, and WVU. Helmi et al. suggested a light-weight feature selection method, called GBOBWO algorithm, for human activity categorization based on a gradient-based optimizer algorithm and a support vector machine-based classifier. They used accelerometer signals as inputs and extract features in a general way [26]. They selected appropriate features based on the GBOBWO method. They achieved 98% accuracy with the UCI-HAR and WISDM database. 

In summary, the accuracy of video and sensor-based behavior identification varies greatly depending on how the properties of the constantly changing input signal are extracted, how segmentation is performed, how features are selected, and how the recognition model is used. As a result, there have been studies conducted to normalize data and extract step-by-step features through multiple CNN layers [24], a study on a method of deriving features that can be distinguished from the background through preprocessing [25], and a study on fusion of various feature values in the signal extraction and selection stage and extracting features of a light input signal that can be operated in a wearable environment [26]. Another study on a method of deriving features that can be distinguished from the A study was also recently undertaken to integrate the picture signal and the acceleration signal and analyze it with a CNN in order to complement the constraints of the input data and thereby increase the accuracy of behavior recognition [23].

However, among existing studies, no study on generalized behavior recognition models that support various types of input signals or minimize the preprocessing of input signals, while also being robust to any noise that may occur in daily life, has been conducted. In addition, in previous studies, deep learning models other than CNN were not applied to fusion. Therefore, in this study, we propose a generalized deep learning model that is robust to noise not dependent on input signals by extracting features through a deep learning model for each heterogeneous input signal that can maintain performance while minimizing preprocessing of, and while integrating, the input signal. In this work, we propose a hybrid deep learning network that can recognize user behavior patterns using heterogeneous signals of image signals and accelerometer sensor signals. We also propose a fusion network that uses two sensors to maximize the identification rate for unfamiliar actions with a single signal and the development of a noise-resistant generalized recognition model. With the proposed model, an image signal and an accelerometer signal are inputted at the same time (for approximately 1 s) in the form of a time-series signal. A ResNet feature is produced for an image signal, and a CNN and CBAM model is used to create a feature for an accelerometer signal. The two signals are then concatenated to identify the activity. 

The technical contribution of this paper is as follows. First, we propose a generalized deep learning model that guarantees the recognition rate even when noise occurs in the acceleration signal or when distortion such as left and right reversal of the image occurs. The suggested model extracts features using a deep learning model that is appropriate for the type of input signal. ResNet is used to extract the features of the image signal, and CNN is used to extract the features of the multi-channel acceleration signal. LSTM and CBMA, which add attention to weights, are used to reflect temporal characteristics. Second, we use heterogeneous input data such as image and sensor data simultaneously to classify human activity, which can maintain performance while minimizing preprocessing of the input signal. Third, with respect to computational time, we offer an optimal input and a model for behavior recognition that takes into account the computational resources and processing time required based on the kind and size of input data. For activity recognition, normalized skeleton data can be enough to classify the actions while maintaining accuracy. The majority of present research has focused on increasing accuracy, although concerns such as training time and processing speed are crucial for practical usage in everyday life. The remainder of this chapter describes the materials, proposed methods, and experiment results in detail.

## 2. Materials and Methods

**Figure 1:**
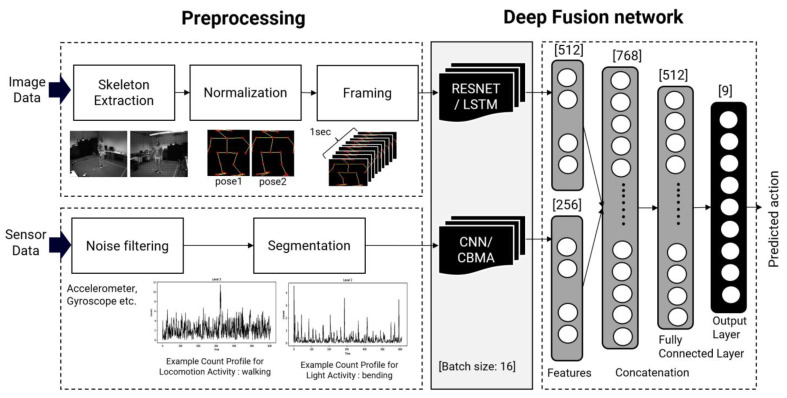
Model architecture.

The proposed method consists of the following procedure, as shown in Figure 1. Overall, all input data are preprocessed, including noise filtering and the segmentation of the accelerometer data. For image data, the skeleton extraction converts the segmented human data into skeleton images through skeleton extraction, normalization, and framing of the image data. Features are then extracted using a different deep learning model. The feature vectors are combined with a fusion network with concatenation and a fully connected layer.

### 2.1. Preprocessing

Each input dataset took each step during the preparation stage. The image signal processing was as follows. In this study, we classified 11 activities, such as jumping in place and clapping hands. We only used 9 activities among the 11 activities because A09 (sitting down) is a combination of A10 (sitting) and A11 (standing up), and it was assumed that sufficient information could be transmitted without discriminating between the two actions in terms of type recognition.

To input consecutively stored image frames for activities, we removed the background from the image based on the background subtraction algorithm. We then extracted 25 joint data from a skeleton detection through OpenPose API [27]. Because they have a feature that can identify activities solely using joint information, joint data were extracted to enhance the speed while maintaining the identification accuracy and classification of the user’s activity. The JSON file format of the person in the frame was used to generate the joint data, which was obtained using the x and y coordinates of the keypoints through a JSON file parser. 

The acquired joint data were normalized through the normalization process after recognizing the skeleton to consider only the parts necessary for activity recognition. In the normalization step, the joint data acquired were scaled to a size of 100 × 100. The normalization process was applied to minimize the differences in individual physical conditions, such as tallness and shortness or bulkiness and smallness, and to improve the learning speed by lightening the data. To reflect the change over time in the input, the data frame was bundled for approximately 1 s to become a single input.

In this study, 20 single-skeleton images were grouped and framed. The reason for creating a frame set by grouping 20 each in the framing process was that the ratio of acceleration data to image data was 1.4:1. In order for the collected data for approximately 1 s of acceleration data to be inputted as input, and for the skeleton image data to also be collected for approximately 1 s to use the data as input, a set of 20 groups was created. 

**Figure 2:**
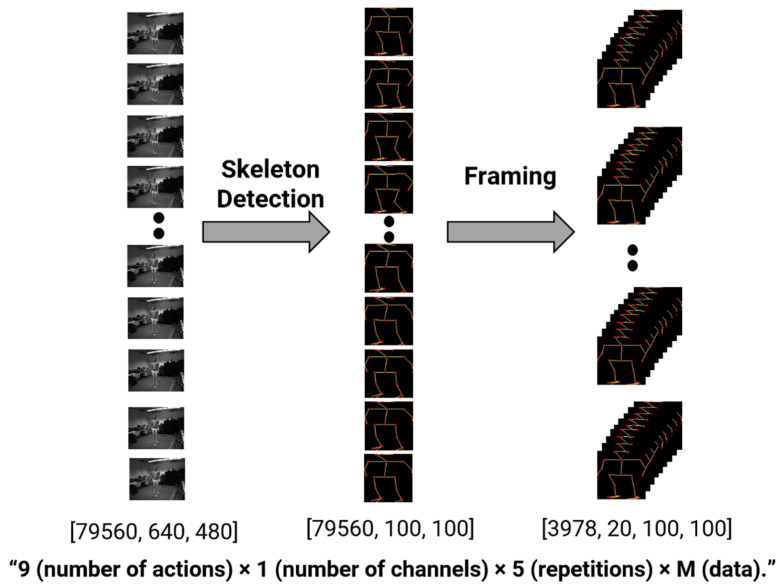
Skeleton image input data for deep learning.

The data format used as the input to the final acquired deep learning model is shown in Figure 2. Each dataset consisted of nine actions, one channel, and five repetitions. After finding the skeleton by inputting sample data with a size of 640 × 480, the size of the input image was reduced, and the image was normalized to a size of 100 × 100 to refine unnecessary information. Framing was then applied to express the flow of time, and an input form of 3978 × 20 × 100 × 100 was finally configured.

**Figure 3:**
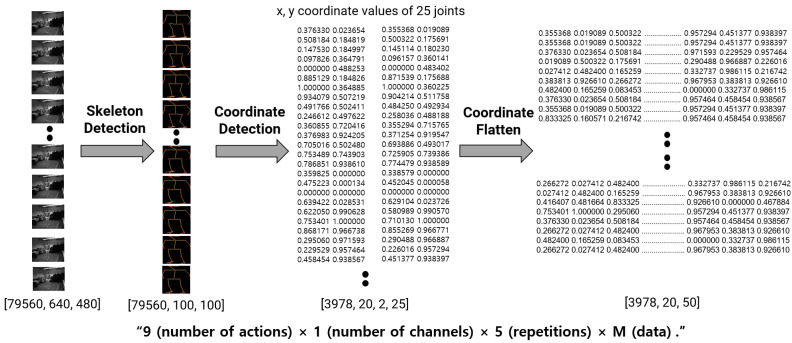
Skeleton coordinate input data for deep learning.

In addition, we used the skeleton coordinate vector as another feature obtained from the image, as shown in Figure 3. After detecting the skeleton, we obtained the joints of each frame and normalized them into 100 × 100-sized images to simplify the input. Each 25-skeleton joint vector was then flattened to input the deep learning model.

**Figure 4:**
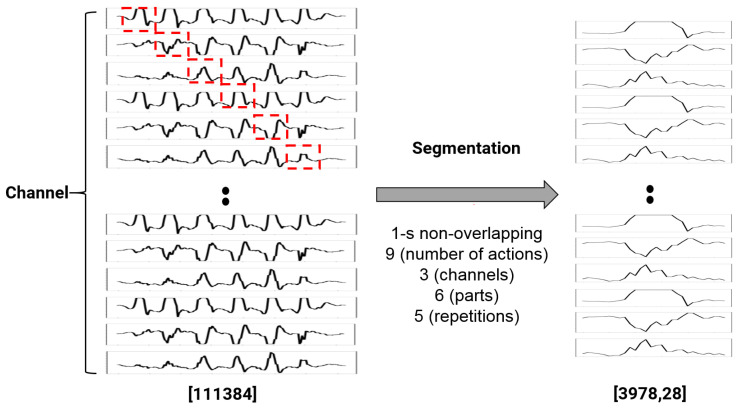
Accelerometer input data for deep learning.

For accelerometer data, the three-axis accelerometer data were measured in 6 bodily locations of 12 subjects: wrists, hips, and feet. After obtaining raw three-axis accelerometer data, we applied bandpass filtering to de-noise the signal, and then segmented the filtered signal into 1 s windows that matched the image frame data. As input, we simultaneously employed continuously collected image data and accelerometer data from the same action. Both signals used 1 s of data as input because the behavior did not consist of a single moment but rather varied over time. We employ a 1 s time-series segmented image frame and an accelerometer signal to classify the time-series activity recognition. For segmentation, we use a 1 s non-overlapping window, as shown in Figure 4. All data consists of 3-channel accelerometer data (x, y, z). As a result, using three axes measured at six locations on the body as each channel, 18 channels were segmented into 28 lengths of approximately 1 s each.

### 2.2. Feature Extraction and Multimodal Data Fusion with Deep Learning

**Figure 5:**
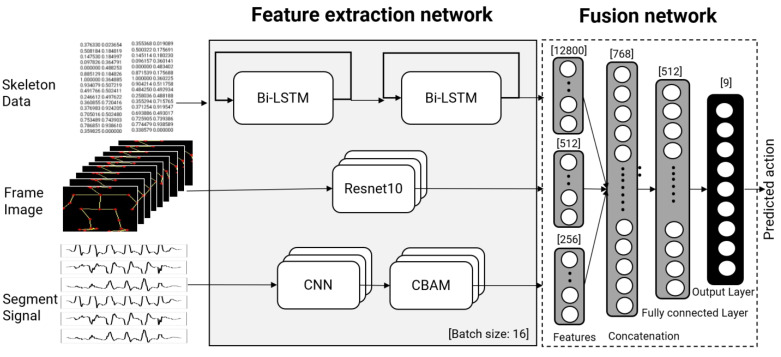
Deep learning model of three input cases.

Features were extracted from each set of input data using the proposed deep learning model. The proposed model architecture is illustrated in Figure 5. We used three different types of inputs in this investigation and built a deep learning model for each input. ResNet10 was used to prevent vanishing gradient problems according to depth for the input images generated by connecting joints, such as the ankles and knees, neck, and shoulder [28]. ResNet10 was used instead of ResNet101 or ResNet151 because the learning results of ResNet10 and ResNet101 did not differ significantly during the test.

In the model using the vector of joint data as input, features were extracted based on the two-layer bidirectional LSTM model. The reason for using the bidirectional LSTM was to learn the difference in the change of the joint vector with the flow of time in the front and the back in a balanced manner [29]. In the case of using only two layers, it was confirmed experimentally that an over-optimization occurred when a deeper layer was used, confirming that two layers were optimized. The joint coordinates finally obtained went through the process of flattening the dimension to be used as an input to the LSTM. Because the coordinate data were extracted from the skeleton image, the number of data points is the same as the number of skeleton images. Therefore, similarly to the skeleton image input, the coordinates obtained were grouped into a single input of 20 data points for classification of a specific length, and not into single data points. Adam was used as the overall optimizer, and the learning rate used to train the model was 0.001. There were 512 feature values from the image processing model. A total of 12,800 features of the skeleton coordinate values were processed using Bi-LSTM.

The preprocessed accelerometer data inputs were used to extract features through the CNN and convolutional block attention (CBAM) models. If the CNN block consisted of two 1d convolutions and two 1d batch normalization parts, batch normalization proceeded after each convolution layer, and after batch normalization, attention was applied through the CBAM attention technique [30]. CBAM attention was largely divided into channel attention and spatial attention and proceeded sequentially. Attention was processed for each channel through the channel attention part, and attention was processed by focusing on where features were located through the spatial attention part. There was a total of 256 feature values of the accelerometer data obtained by processing the CNN and CBAM models. The CNN model consists of two convolutional layers, and the first convolutional layer consists of 128 filters with a filter size of 3. After the convolution layer, batch normalization and an ReLU function were applied, and the CBAM attention model was then applied. If the second convolutional layer consisted of 256 filters with a filter size of 3, batch normalization and the ReLU function were applied as in the first step, and the CBAM attention model was then applied. CBAM stands for the convolutional block attention module as an attention module to which the self-attention technique is applied. The CBAM is an improved module for BAM attention. BAM uses the attention obtained through the parallel processing of the channel attention and spatial attention, whereas CBAM obtains the context and local information by sequentially applying channel attention and spatial attention.

The feature set from ResNet10 and the feature set from the CNN+CBAM model were integrated to classify the activities through the fully connected layer and the output layer. The batch size of the deep learning model was 16, and the input size of the feature stage was 512 for the images and 256 for the accelerometer signals. The number of nodes in the concatenation stage was 768, the number of nodes in the fully connected layer was 512, and the model classified the 9 activities. The hyperparameters used in this study are listed in Table 1. The hyperparameters were determined according to the type of the proposed deep learning fusion model, and the parameters showing the best performance in each case are as follows: The hyperparameters were determined according to the type of the proposed deep learning fusion model. The best overall performance was achieved when ResNET10, CNN, and CBAM were combined, and the learning rate was set to 0.001, the batch size to 4, and the epoch to 36. The epoch is distinct since it is a result of the process of determining an optimum parameter based on the training and validation losses. The magnitude of the input data is related to the substantial difference in epochs depending on the model. The size of the input data in the ResNet10+ Bi-LSTM+CNN+CBAM model, which incorporates all input data, was as follows: (3978, 20, 100, 100) is the skeleton data matrix, (3978, 20, 50) is the coordinate, and (3978, 28) is the accelerometer data. As a result, 1 epoch time for training, or the time it takes to train the model, was the longest at 300 s, while the number of epochs, or the iteration for learning the full model, was the shortest at 16. Conversely, the data size was the smallest when vector data and sensor were utilized, but the training time was 10 s and the epoch size was 135. That is, when the input data to be trained was small, it was confirmed that the training time was reduced, but the iterations to find the optimized model increased.

## 3. Results

### 3.1. Dataset

The dataset used was Berkeley MHAD [31]. The data used in this dataset were Multiview video and acceleration data. The multi-view video data were in a 640 × 480 pgm file and consisted of 12 camera data points. We used 4 out of the 12 camera data points. The data from the 12 cameras were divided into three categories: data from the front, data from above, and data from the back. In this work, we used data from the front camera to reduce the number of cameras in order to use this study in everyday life. The degree of generalization of the model was proven using image inversion experiments (left-right inversion and up-down inversion). In this dataset, 11 actions were repeated 5 times by 7 males and 5 females aged 23–30. There were approximately 660 sequences in total. Table 2 lists the 11 action lists used and the number of samples. We used 9 of the 11 activities because A09 (sitting down) is a combination of (A10 sitting) and (A11 standing up), and it was assumed that sufficient information could be transmitted without discriminating between the two actions in terms of type recognition. 

The acceleration data consisted of six three-axis wireless accelerometers measuring the wrist, ankle, and hip movements. The accelerometer signal data were obtained from the three-axis accelerometer sensor data from six places on the body, i.e., both wrists, both hips, and both feet, from the 12 subjects. The accelerometer signal input data points numbered 111,384, which included nine activities, three channels, six parts, and five repetitions. The skeleton data consisted of nine actions, one channel, and five repetitions. After finding the skeleton by inputting 79,560 sample data with a size of 640 × 480 as the input, the size of the input image was reduced, and the image was normalized to a size of 100 × 100 to refine the unnecessary information. Framing was then applied to express the flow of time, and the input form of 3978 × 20 × 100 × 100 was finally configured. To achieve a lower storage capacity than the image data, the joint coordinate data were encoded as a vector. The coordinate values of the 25 joints were expressed in real integers before being flattened into a single numeric vector. Finally, a 3978 × 20 × 50 input vector was constructed in the form of nine actions with one channel, which were repeated five times. 

Joint data were acquired from a human image based on the OpenPose program [27]. We used 25 joint points, as shown in Table 3. Joint data were acquired from the segmented human image based on the Open Pose program. The joint data were composed of a JSON file format of the person in the frame, which was acquired through the x- and y-coordinates of the keypoints through the JSON file parser. In the normalization step, the acquired joint data were scaled to a size of 100 × 100. The normalization process was conducted to minimize the differences in the individual physical conditions, such as tallness and shortness and bulkiness and smallness, and to improve the learning speed by lightening the data.

### 3.2. Experiment Results

The overall accuracy of recognizing the nine activities was 70.9% using only image data, 84.7% using only the accelerometer in the model, 94.8% using a skeleton image and accelerometer sensor data, 91.8% using skeleton coordinate vector and accelerometer data, and 93.1% using all three input data points as shown in Table 4. When skeleton image data and an acceleration sensor were used as inputs, the overall behavior recognition performance was confirmed to be the best. When using the skeleton image and sensor data to calculate the difference in accuracy for each activity, the standard deviation for each activity was approximately 5%. The standard deviations were 14% and 11% when the image or sensor data were used as inputs. This demonstrates that when both images and sensor data are used, all types of behavior can be recognized equally.

**Figure 6:**
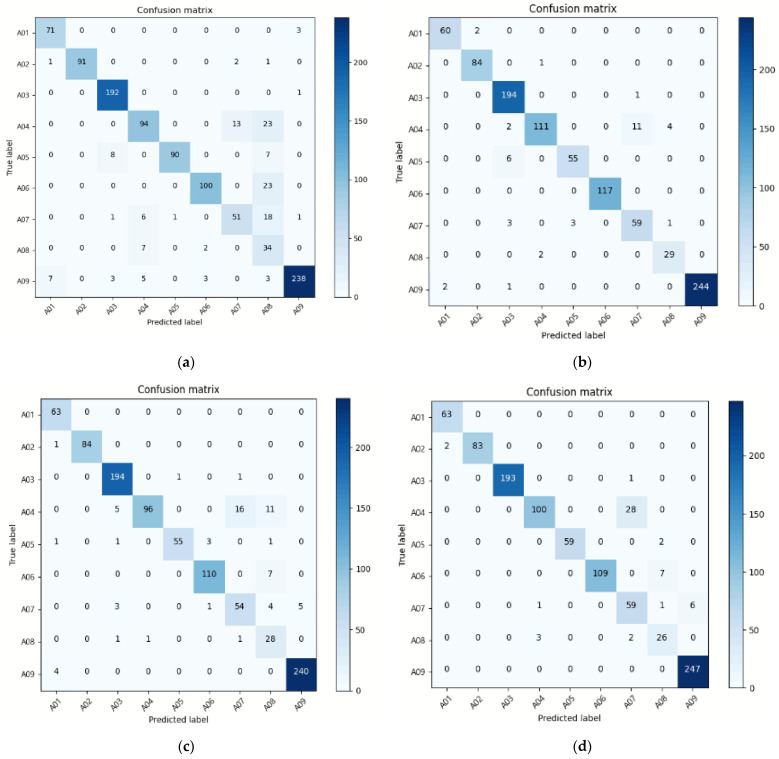
Confusion matrix of activity classification: (**a**) sensor-only confusion matrix, (**b**) accelerometer and skeleton image, (**c**) skeleton coordinates and accelerometer, and (**d**) accelerometer, skeleton vector, and skeleton image.

When only the sensor was used, the accuracy was approximately 85%, and when the ResNet-based prediction applying only an image was used, the accuracy was approximately 70%. However, the analysis using the accelerometer and image information confirmed an accuracy of 94.8%. As a result of the analysis using the accelerometer and coordinate information, it was 91.8%. As a result of the analysis using an accelerometer, image information, and coordinate information, the accuracy was 93.1%. Through the confusion matrix, we examined which behavioral analyses did not work well for a more extensive investigation, as shown in Figure 6. The relative color indicates the relative accuracy in confusion matrix. We found that when both images and sensor data were used, the accuracy of each behavior recognition was higher than when only sensors or image data were used in most circumstances. However, all classes that are difficult to categorize are those that include the use of the hands and arms. In the case of the sensor-only model, A04 (punching), A05 (waving—two hands), A06(waving—one hand (right)), A07 (clapping hands), and A08 (throw balls), which are most of the actions using hands, had low individual classification accuracy and were difficult to classify because of the similarities among the data, as shown in Figure 6a. When the sensor and image data were used together, it was confirmed that the performance of distinguishing A04, A07, and A08 was improved, as shown in Figure 6b–d. A04 and A07 were difficult to differentiate in the image because they both comprised stretched hands in a standing position, with no leg movement in the sensor data, making it difficult to distinguish them solely on the basis of identical hand movements.

**Figure 7:**
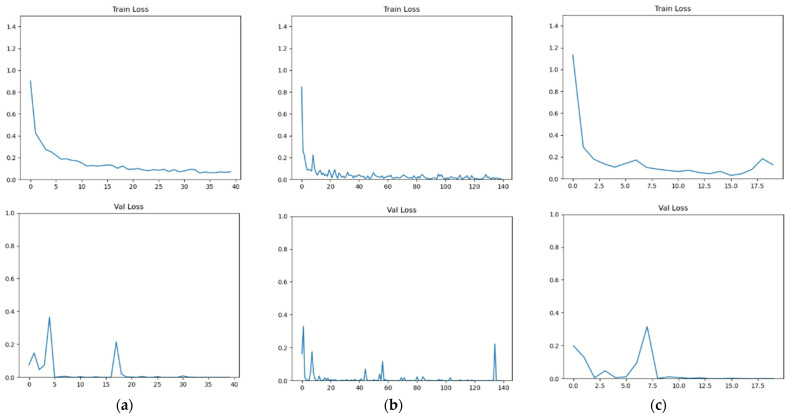
Train loss graph: (**a**) accelerometer and skeleton image with ResNet10+CNN+CBAM, (**b**) skeleton coordinates and accelerometer with Bi-LSTM+CNN+CBAM, and (**c**) accelerometer, skeleton vector, and skeleton image with the ResNet10+ Bi-LSTM+CNN+CBAM model.

The performance, analyzed in terms of time is shown in Figure 7 and Table 1. Figure 7 shows the train loss and validation loss of our proposed method. To avoid overfitting, we used train loss and validation loss to generate optimal parameters. The moment where the train loss converges to 0 and the validation loss converges was chosen as the stopping criterion as the overall accuracy improves. We measured the time it takes for one epoch to train to evaluate performance over time, as shown in Table 1. For skeleton and accelerometer data using the ResNet10+CNN+CBAM model, one epoch took approximately 275 s. It took 10 s to apply Bi-LSTM+CNN+CBAM with skeleton coordinate data and acceleration data as inputs under the same circumstances. It took 300 s to take all three forms of data and apply them to the ResNet10+ Bi-LSTM+CNN+CBAM model. The size of the input data in the ResNet10+ Bi-LSTM+CNN+CBAM model, which incorporates all input data, was as follows: (3978, 20, 100, 100) is the skeleton data matrix, (3978, 20, 50) is the coordinate, and (3978, 28) is the accelerometer data. This indicates that 1 epoch time for training, or the time it takes to train the model, was the longest at 300 s. Conversely, the data size was the smallest when vector data and sensor were utilized, but the training time was 10 s. That is, when the input data to be trained was small, it was confirmed that the training time was reduced, but the iterations to find the optimized model increased.

**Figure 8:**
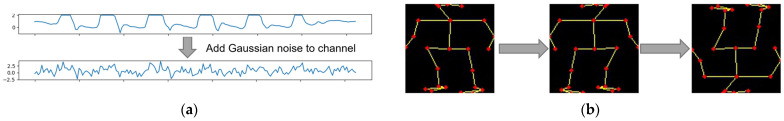
Input data distortion: (**a**) adding Gaussian noise to one channel of an accelerometer sensor and (**b**) a flipped image from left to right, bottom to top.

Finally, in this study, a performance analysis was conducted from the perspective of model robustness when various noises were added to the input signal. In the case of behavior recognition achieved through images, if the angle of the camera is changed, a performance degradation may occur during testing. We generated two kinds of input signal noise, as shown in Figure 8. First, we tested the input data with Gaussian noise added to 1, 3, and 6 channels among 18 channels to the acceleration data. Second, in order to add noise to the image data, the image was used as input to the model by inverting the image left and right and up and down.

**Figure 9:**
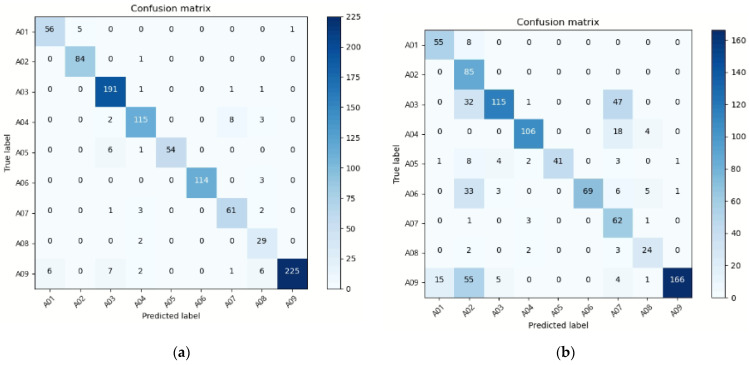

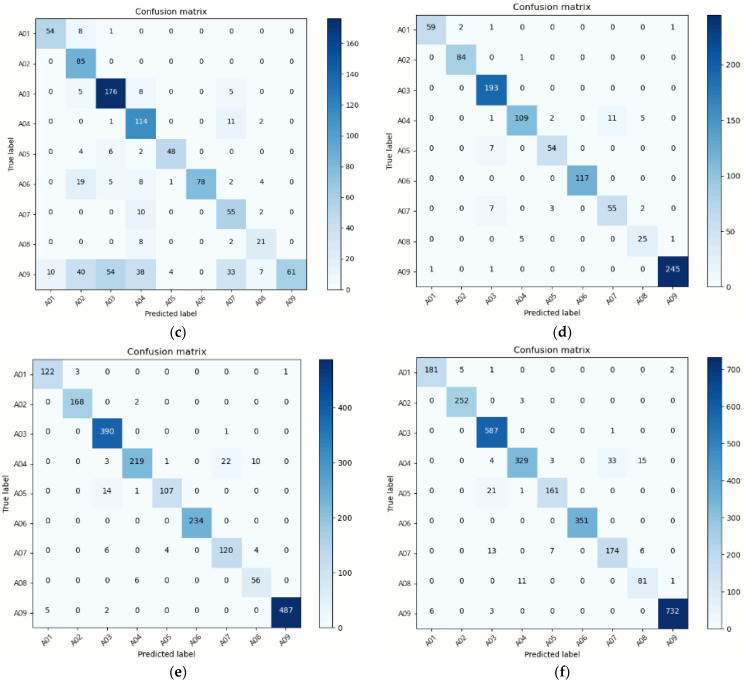
Confusion matrix of activity classification: (**a**) 1 channel sensor data distortion (0.5% noise), (**b**) 3 channels sensor data distortion (16.7% noise), (**c**) 6 channels sensor data distortion (33.3% noise) (**d**) image data distortion (normal and left-right reverse data), (**e**) image data distortion (normal and top-bottom reverse data), (**f**) image data distortion (normal and left-right reverse and top-bottom reverse data).

Among the 18-channel acceleration data, all 1-channel, 3-channel, and 6-channel values were replaced with Gaussian noise, as shown in the Figure 9a–c. Through the experiment, it was confirmed that the performance was affected as the number of channels with errors increased. When noise was added to 1 channel, around 0.5% of noise was formed, 16.7% of noise was generated on 3 channels, and 33.3% of noise was generated on 6 channels. As a result of the experiment, the accuracy was 94.8% while using a skeleton image and sensor as an input, but 93.8% when noise was generated in one channel, confirming that the performance degradation was 1%. The overall accuracy was 77.1% when noise was applied to three channels, and 76.3% when noise was added to six channels. It was confirmed that when the channel noise was 0.5% or above, the performance of the acceleration signal degraded dramatically. In addition, the ninth action, sitting down, exhibited a considerable decline in performance dependent on the channel distortion, as indicated in the confusion matrix. As shown in Figure 9d–f, when the image in which the input signal was inverted toward the left and right was mixed and tested with the existing image, the analysis showed 93.41% accuracy, and when tested by mixing the vertically inverted image and the existing image, the performance was 94.16%. In the case of mixing the left and right images, the upside-down image, and the existing image, a good performance of 93.44% was demonstrated.

**Figure 10:**
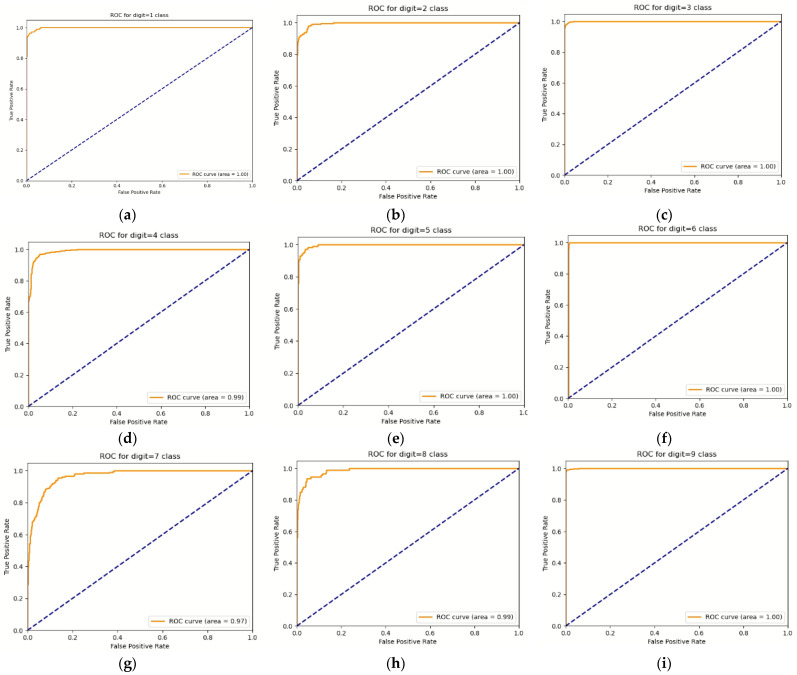
ROC curve and AUC value for each activity class. (**a**) A01 class, (**b**) A02 class, (**c**) A03 class, (**d**) A04 class, (**e**) A05 class, (**f**) A06 class, (**g**) A07 class, (**h**) A08 class, (**i**) A09 class.

Figure 10 shows the ROC (receiver operating characteristic) plot and AUC (area under ROC curve) value of the result of classifying the behavior using the skeleton image and sensor data. In most classes, the AUC value was 1, except for A04, A07, and A08. The AUC of A04 was 0.99, the AUC of A07 was 0.97, and the AUC of A08 was 0.99. In general, a classifier is considered good if its AUC is 0.95 or higher. Because all of the classifiers in this study had a score of 0.95 or above, it can be concluded that the proposed strategy has produced an efficient classifier. In the case of the ROC plot, it shows good classifier performance in most classes. It was confirmed that the classifier performance did not decrease significantly even in the case of A07 and A08, which had relatively low accuracy.

## 4. Discussion

In the case of deep-learning-based recognition, a robust deep learning model cannot be generated if there are insufficient data or if the activity to be recognized is deformed. For example, if the image is rotated, it is not possible to recognize whether the image has been rotated, and the possibility of determining a different posture increase. That is, even in the case of the same standing action, there is a problem in that a different pose can be recognized when lying down or standing. To solve this problem, a study was conducted to generate learning data by applying a self-supervised learning technique to augment a small amount of data [19,20].

From this study, we observed that the proposed deep learning model preserves the recognition rate even when various poses are inputted for confirming experimentally the complementary behavior recognition of the accelerometer and image sensor. The overall performance was demonstrated by establishing a deep fusion network with heterogeneous inputs, and the fluctuation in the recognition rate for each behavior was also reduced. Boxing or punching movements, for instance, have a similar pattern of arm motions and arm bending, which are comparable to those of clapping and throwing a ball. As a result, when only an image signal was used, the accuracy was only approximately 50%; however, when both the image and the sensor signal were used, the accuracy was determined to be approximately 86%.

In addition, when noise or distortion occurs in image or sensor data, it was confirmed that the proposed system can recognize the behavior while maintaining accuracy in the absence of noise. The accuracy analysis indicated a 93.41% performance when the image with the input signal was inverted toward the left or right and the existing image was mixed and evaluated, and a 94.16% performance when the vertically inverted image and the existing image were mixed and tested. The system performed well when merging the left and right images, the upside-down image, and the current image, reaching an accuracy of 93.44%. White noise was also added to one channel value in the case of the acceleration signal, and the test confirmed that the performance was 93.23%. After testing the data with inversion and noise data, it has been determined that the suggested model is resilient, with a performance deterioration of approximately 1%.

By comparing the performance with the training time, we confirmed that activity recognition above a certain level is possible if only skeleton data are needed for such recognition. In terms of a simple temporal efficiency, the model using sensor and coordinate information showed a good performance, after which the accelerometer and image model and the model using accelerometer, image, and coordinate information showed similar performance. Instead of using the complete image as the input for behavior recognition, the proposed method employs a skeleton image and its coordinate values. This reduces both learning time and learning accuracy. When all data were analyzed at the same time, it took approximately 300 s to learn the three proposed deep learning networks, but only approximately 10 s when the skeleton coordinate vector was utilized as an input. As a result, a performance improvement of approximately 30-fold in terms of time efficiency was confirmed.

The limitations of the proposed study are as follows. The deep learning model proposed in this study applied ResNet for image data, CNN and CBAM for time series signals, and LSTM for skeleton vector data to apply a model suitable for input signals. This model is widely used in the deep learning field, and it seems that additional performance improvements can be expected. Especially, the existing skeleton vector produces poor results. Additional speed increases can be predicted if one employs a learning method that weighs coordinate changes based on a transformer or BERT model used for sequential data modeling instead of LSTM. In addition, to optimize performance and processing power, a skeleton, not an original image, was extracted and used as an input. However, considering that research on generalizing models based on limited input signals in recent studies is ongoing, further research on end-to-end models based on raw input signals is needed. Lastly, behavior recognition was performed using current 18 channel data extracted from six wearable devices and camera images in four directions. More research is needed to figure out how to simplify the input signal and put it to use in the actual world.

## 5. Conclusions

In this study, we proposed a hybrid deep learning model that uses heterogeneous sensor data, an acceleration sensor, and an image as inputs. We used CNN and CBAM models for the accelerometer data, and bi-LSTM and ResNet10 for the image data. From the analysis of nine behaviors using the Berkeley MHAD dataset, the overall accuracy was 94.08% with a skeleton image and accelerometer data, and 93.09% with a skeleton image, the coordinates, and accelerometer data. In addition, when the image in which the input signal was inverted toward the left or right was mixed and tested with the existing image, the analysis showed an accuracy of 93.41%, and when tested by mixing the vertically inverted image and the existing image, the performance was 94.16%. In the case of mixing the left and right images, the upside-down image, and the existing image, a good performance of 93.44% was demonstrated. In addition, in the case of the acceleration signal, white noise was added to one channel value, and the test results confirmed that the performance reached 93.23%. As a result of testing using inversion and noise data added, it has been confirmed that the proposed model is robust, with a degradation in performance of only approximately 1%. This enables a stable behavior recognition even when various poses are changed or when noise is applied to the sensor data.

## Figures and Tables

**Table 1:** Hyperparameters of deep learning models.

Training Models		ResNet10+ Bi-LSTM+ CNN+CBAM	ResNet10+ CNN+CBAM	Bi-LSTM+ CNN+CBAM
Input type		Image + vector + sensor	Image + sensor	vector + sensor
Training parameters	Learning rate	0.001	0.001	0.001
	Batch size	16	4	16
	Epoch size	13	36	135
Performance measures	1 epoch time ^1^	300 s	275 s	10 s
	Overall accuracy	93.1%	94.8%	91.8%

^1^ One epoch time for training.

**Table 2:** Activity list.

Code	Action Description	# of Instances
A01	Jumping in place	6025
A02	Jumping jacks	7824
A03	Bending—hands up all the way down	18,762
A04	Punching (boxing)	10,195
A05	Waving with two hands	9742
A06	Waving one hand (right)	10,763
A07	Clapping hands	5242
A08	Throwing a ball	3584
A09	Sitting down and standing up	21,086
A10	Sitting down	6025
A11	Standing up	7824

**Table 3:** Joint data from skeleton detection.

Index	Part	Index	Part
0	Nose	13	Left Knee
1	Neck	14	Left Ankle
2	Right Shoulder	15	Right Eye
3	Right Elbow	16	Left Eye
4	Right Wrist	17	Right Ear
5	Left Shoulder	18	Left Ear
6	Left Elbow	19	Left Big Toe
7	Left Wrist	20	Left Small Toe
8	Mid Hip	21	Left Heel
9	Right Hip	22	Right Big Toe
10	Right Knee	23	Right Small Toe
11	Right Ankle	24	Right Heel
12	Left Hip	25	Background

**Table 4:** Activity recognition accuracy.

Activity	Code	# of Instances	Accuracy (%)				
			Image Only	Sensor Only	Image + Sensor	Vector + Sensor	Image + Vector + Sensor
Jumping in place	A01	6025	53%	95%	96%	100%	100%
Jumping jacks	A02	7824	67%	95%	98%	98%	97%
Bending—hands up	A03	18,762	86%	99%	99%	98%	99%
Punching (boxing)	A04	10,195	56%	72%	86%	75%	78%
Waving—two hands	A05	9742	70%	85%	90%	90%	96%
Waving—one hand (right)	A06	10,763	69%	81%	100%	94%	93%
Clapping hands	A07	5242	48%	65%	89%	80%	88%
Throwing a ball	A08	3584	49%	79%	93%	90%	83%
Sitting down, then standing up	A09	21,086	83%	91%	98%	98%	100%
Average accuracy			70.9%	84.7%	94.8%	91.8%	93.1%
Standard deviation of activity accuracy			14%	11%	5%	9%	8%

## Data Availability

Berkeley MHAD. Available online: https://tele-immersion.citris-uc.org/berkeley_mhad (accessed on 21 December 2021).
